# What drives weight status among female university students? A machine learning analysis of sociodemographic, dietary, and lifestyle determinants

**DOI:** 10.3389/fnut.2025.1574063

**Published:** 2025-07-18

**Authors:** Radwan Qasrawi, Abir Ajab, Leila Cheikh Ismail, Ayesha Al Dhaheri, Sharifa Alblooshi, Razan Abu Ghoush, Stephanny Vicuna Polo, Malak Amro, Suliman Thwib, Ghada Issa, Haleama Al Sabbah

**Affiliations:** ^1^Department of Computer Science, Al-Quds University, Jerusalem, Palestine; ^2^Department of Computer Engineering, Istinye University, Istanbul, Türkiye; ^3^Department of Clinical Nutrition and Dietetics, College of Health Sciences, University of Sharjah, Sharjah, United Arab Emirates; ^4^Nuffield Department of Women’s & Reproductive Health, University of Oxford, Oxford, United Kingdom; ^5^Department of Nutrition and Health, College of Medicine and Health Sciences, UAE University, Al Ain, United Arab Emirates; ^6^Department of Health Sciences, College of Natural and Health Sciences, Zayed University, Dubai, United Arab Emirates; ^7^Department of Public Health, College of Health Sciences, Abu Dhabi University, Abu Dhabi, United Arab Emirates

**Keywords:** body mass index, dietary patterns, lifestyle behaviors, machine learning, obesity, weight management

## Abstract

**Background:**

Obesity and underweight are increasingly common among young adult women, often resulting from complex interactions between diet, lifestyle, and socioeconomic factors. This study addresses that gap by applying machine learning to a wide range of behavioral, dietary, and demographic data. The main research question asks: What are the key factors influencing weight status among female university students, and how accurately can machine learning models identify them? We hypothesize that different factors are significantly associated with underweight, overweight, and obesity, and that machine learning can reliably detect these patterns. The aim is to identify the strongest predictors and support more targeted weight management strategies.

**Methods:**

This cross-sectional study analyzed data from 7,092 female university students (aged 18–30 years) in Palestine and the UAE. Sociodemographic, dietary, and lifestyle predictors were evaluated using machine learning (Random Forest, SVM, logistic regression, gradient boosting, decision trees, and ensemble methods). Synthetic Minority Over-sampling (SMOTE) addressed class imbalance. Model performance was assessed via 10-fold cross-validation, with significance determined by the chi-square test (*p* < 0.05, 95% CI).

**Results:**

The Random Forest model achieved the highest accuracy (obesity: 96.8%, underweight: 94.6%, overweight: 90.3%) and AUC (0.95–0.97). The main drivers of weight status categories were as follows: underweight was associated with low water/milk intake and preference for fast food; overweight with added oil, large eating quantity, and low physical activity; and obesity with energy drink consumption, salty snacks, and irregular meals. All findings were statistically significant (*p* < 0.001). Socio-demographic factors (e.g., low income and marital status) and lifestyle habits (e.g., sleep <5 h and fast eating) were also significantly related to weight status.

**Conclusion:**

The integration of these findings into weight management frameworks can significantly enhance the detection and understanding of modifiable determinants, thereby informing public health interventions, guiding the development of targeted weight management strategies, and contributing to the global movement toward healthier bodies.

## 1 Introduction

Obesity and weight status are escalating public health challenges worldwide, contributing to a range of chronic conditions, including diabetes, cardiovascular disease, and certain cancers, and imposing substantial burdens on healthcare systems (1, 2). While non-modifiable factors such as genetics and family history influence weight status, modifiable behaviors, especially dietary patterns and lifestyle choices, play a critical role in determining an individual’s body mass index (BMI) (3, 4). Accordingly, identifying and integrating these modifiable risk factors are essential for developing effective interventions aimed at preventing and managing unhealthy weight trajectories across populations (5, 6).

In recent years, healthcare provision has begun shifting from a general-purpose approach toward personalized precision health, which uses individual characteristics to customize prevention and treatment strategies (7–9). Personalized precision health focuses on adapting healthcare interventions to the personal characteristics and needs of individual patients (9). One critical aspect of personalized precision health is the prediction and management of weight status, a key determinant of overall health and wellbeing (10). This evolution has been primarily driven by advances in technology, particularly in the areas of machine learning (ML) and artificial intelligence (AI) (11), which offer powerful tools to analyze complex, high-dimensional data and uncover non-linear relationships among risk factors (9–12). By incorporating a broad range of clinical, sociodemographic, and behavioral variables into predictive models, ML techniques can enhance the accuracy of weight status predictions and support the design of targeted, data-driven weight management programs (13–15).

Recent literature demonstrates the promise of ML in obesity research at multiple life stages. One study focusing on early childhood used electronic health records and ML algorithms to predict obesity risk, enabling timely preventive measures (16). In adults, various ML methods, including support vector machines, Random Forest, and gradient boosting, have been applied to large survey and epidemiological datasets to identify key predictors of overweight and obesity (17).

Additional studies emphasized weight management as an essential strategy for combating obesity and related disorders, including lifestyle changes, dietary adjustments, and the use of AI-based meal recommendation systems (18, 19). Optimizing machine learning for weight status prediction is particularly appealing due to its capacity to incorporate a wide range of clinical and lifestyle-related variables into predictive models (20). Recent research has emphasized the significance of considering multiple factors when addressing weight-related concerns. Elements such as physical activity, dietary choices, sleep quality, and stress levels collectively influence an individual’s weight status (10, 14, 21).

Despite these advances, many existing models concentrate on isolated predictors or lack comprehensive integration of lifestyle, dietary, and sociodemographic factors (21, 22). Consequently, there remains a need for holistic predictive frameworks that not only classify weight status but also identify the most influential modifiable factors driving transitions between the underweight, normal weight, overweight, and obese categories.

In this study, the gap is addressed by using ML classifiers, including support vector machines, Random Forest, logistic regression, gradient boosting, decision trees, and ensemble methods, to analyze a synthetic dataset of 7,092 young adult women from Palestine and the UAE. The target variable was set as weight status (underweight, healthy, overweight, or obese) and all other input values work to predict the classification accordingly. Feature selection tests are then performed to determine which factors have the most predictive influence on weight group classification (22, 23). This study aims to develop and validate a comprehensive machine learning-based predictive model that identifies and ranks the most influential sociodemographic, lifestyle, and dietary factors associated with weight status in young adult women, with the objective of guiding personalized weight management strategies.

## 2 Materials and methods

### 2.1 Data source

#### 2.1.1 Study settings

To gather study data, the authors used a cross-sectional survey design involving female students from Zayed University and Sharjah University in the United Arab Emirates (UAE), as well as Al-Quds University in Palestine (https://emfid.org/zayed/).

#### 2.1.2 Study period

The data collection was carried out from August 2023 to January 2024, capturing a representative sample during this timeframe.

#### 2.1.3 Study population

Participants in the study comprised national and expatriate students, particularly in the UAE, who fully participate in the Palestinian or Emirati cultures. Recruitment efforts were undertaken at both institutions using classroom announcements and advertisements. The ultimate goal was to ensure a balanced representation of weight statuses, using the body mass index (BMI). The authors aimed to acquire a dataset that encompassed individuals across underweight, healthy, overweight, and obese weight ranges (24).

#### 2.1.4 Inclusion and exclusion criteria

To be included in the study, participants were required to meet several criteria. They had to be female and between the ages of 18 and 30 years. Additionally, they needed to be enrolled at Zayed University or Sharjah University in the United Arab Emirates (UAE) or Al-Quds University in Palestine. Participation was contingent upon providing informed consent, and individuals had to be generally healthy, with no chronic illnesses that could influence their dietary habits or body weight.

Several exclusion criteria were also applied. Male students and individuals who were either younger than 18 or older than 30 years were not eligible to participate. Students not enrolled at Zayed University, Sharjah University, or Al-Quds University in Palestine were excluded, as were those who did not provide informed consent. Participants diagnosed with chronic illnesses or medical conditions that could significantly affect their diet or weight were also excluded. Furthermore, pregnant students and those with incomplete or missing data on key study variables were excluded from the study.

#### 2.1.5 Sample size and sampling procedures

The initial dataset consisted of 680 participants, presenting a notable class imbalance that could negatively impact model performance. To address this, we applied the Synthetic Minority Over-sampling Technique (SMOTE) *exclusively to the training set* after splitting the data into training (70%), testing (20%), and validation (10%) subsets. SMOTE generates synthetic samples for the minority class by interpolating between existing instances, thereby enhancing class balance without duplicating records (25, 26).

This oversampling increased the size of the training set, allowing the machine learning models to learn from a more representative and balanced dataset. Importantly, the test and validation sets were left untouched, preserving their original distribution and ensuring an unbiased evaluation of the models. The final training data size, after SMOTE, was expanded to 7,092 records, which was limited to model training only.

Following oversampling, the training data distribution was assessed and confirmed to approximate normality, which supports the assumptions of subsequent statistical procedures. Recognizing that SMOTE can introduce potential risks such as overfitting or artificial decision boundaries, we used 10-fold cross-validation during training to monitor model generalizability and prevent overfitting (see Section 2.5).

Additionally, all model hyperparameters were fine-tuned within the cross-validation framework using only the training data, as detailed in Section 2.4. This approach ensured that our model development process remained free from data leakage and provided reliable performance estimates based on unseen data.

#### 2.1.6 Data collection and quality assurance

The questionnaire used for data collection was developed based on established tools and literature relevant to dietary habits, lifestyle behaviors, and sociodemographic characteristics. It was initially drafted in English, then translated into Arabic and back-translated to ensure linguistic accuracy. A pilot study was conducted with a sample of 40 participants from the target population to evaluate the questionnaire’s clarity, readability, and cultural relevance. Based on the feedback, minor adjustments were made to improve question phrasing and overall flow. Content and face validity were assessed by a panel of experts in nutrition, public health, and behavioral sciences. Internal consistency was evaluated using Cronbach’s alpha, confirming the reliability of the instrument. Prior to data collection, all field researchers received standardized training by the principal investigators on ethical procedures, interview techniques, and survey administration. The data collection process was closely supervised to ensure quality, consistency, and adherence to the study protocol.

This study was conducted in accordance with the principles of the Declaration of Helsinki, and all procedures involving human participants were approved by the Zayed University Ethical Committee (Approval No. ZU20\_163\_F). Before data collection began, all participants provided informed consent through a detailed consent form that explained the study’s purpose, procedures, potential risks, and expected benefits. Participants then completed a structured questionnaire online, which collected data on demographics, lifestyle habits, and dietary behavior.

To ensure data reliability, pre-tested questionnaires were used, and participants received clear instructions on how to complete them. Incomplete responses or logically inconsistent answers were identified and excluded.

### 2.2 Feature selections

The study collected a thorough set of features generally related to body weight, body composition, dietary patterns, and food preferences and consumption. These variables served as predictor variables for the target, the weight group. Participants were categorized into these groups based on their Body Mass Index (BMI), with underweight defined as BMI < 18.5, normal weight as BMI 18.5–24.9, overweight as BMI 25–29.9, and obese as BMI ≥ 30. A breakdown of the features is provided below and further illustrated in Table 1.

- Assessing Body Weight and Body Composition: This category of features includes essential measurements related to participants’ physical characteristics. It encompasses variables such as body weight, measured in kilograms, which provides insight into participants’ overall mass and height, measured in meters, and offers information about their stature.
- Physical Activities: The physical activity was classified into three categories. Highly Active: Responds “Yes” to both Question 1 and Question 2, indicating that the individual meets or exceeds the recommended levels of both moderate and vigorous physical activities. Moderately Active: Responds “Yes” to Question 1 but “No” to Question 2, indicating that the individual meets the recommended levels of moderate physical activity but not vigorous physical activity, or responds “Yes” to Question 2 but “No” to Question 1, indicating that the individual meets the recommended levels of vigorous physical activity but not moderate physical activity. Low Activity: Responds “No” to both Question 1 and Question 2 but “Yes” to Question 3, indicating that the individual engages in both moderate and vigorous activities but does not meet the recommended levels for either. Inactive: Responds “No” to all three questions, indicating that the individual does not meet the recommended levels for either moderate or vigorous physical activities.
- Understanding Dietary Patterns: These features explored participants’ dietary habits and preferences. A validated Food Frequency Questionnaire (FFQ) was used to assess their dietary patterns (27). The frequency of food consumption is categorized as never, daily, weekly, or monthly, along with a measure of daily consumption in cups of specific items such as milk and juice, among others. Participants’ preferences for various food items are measured on a three-level scale (do not like, like, or like a lot). This information provides valuable details about their food choices, including fruits, vegetables, legumes, meats, snacks, beverages, and cultural food items (28).
- Fast-Food Consumption: This category focuses on the frequency of fast-food consumption among participants. Participants are asked to indicate how often they consume specific fast-food items, with options ranging from never to twice to four times a week (29). Fast-food items assessed include burgers, fried chicken, fries, pizza, shawarma, chips, and noodles. This data provides information on participants’ fast-food consumption habits, allowing for the assessment of their regularity in consuming these items.

**Table 1:** Description of the features in the machine learning models.

Category	Features
Assessing body weight	Body weight (kg), height (m)
Understanding dietary patterns	Dietary habits assessed using FFQ^*^ include food consumption patterns (never, daily, weekly, monthly), daily consumption (cups) for specific items, and food preferences (do not like, like, like a lot) for various food items.
Fast food consumption	Frequency of consuming fast-food items (never, 1–2 times a month, less than four times a month, once a week, 2–4 times a week) for: burgers, fried chicken, fries, pizza, shawarma, chips, and noodles.
Lifestyle variables	Moderate physical activity: Do you do moderate physical activity (activities that take moderate physical effort and make you breathe somewhat harder than normal) at least 150 to 300 min per week? “No” or “Yes.” Vigorous physical activity: Do you do vigorous physical activity (activities include heavy lifting, digging, aerobics, or fast bicycling, etc.) at least 75–150 min per week? “No” or “Yes.” Combination of moderate and vigorous physical activity: Do you do both moderate physical activities less than 150 min, and vigorous activity, less than 75 min per week? “No” or “Yes.”
Eating habits	Regular lunch, regular dinner, daily breakfast, breakfast on the weekend, eating speed, and drink water
Sociodemographic variables	Gender, age, education level, income level, marital status, and nationality
Food allergy	Include reactions to peanuts, tree nuts (such as almonds, walnuts, and cashews), milk, eggs, wheat, soy, and fish, or any specific food.

*FFQ, food frequency questionnaire.

### 2.3 Statistical and ML analysis

Before applying machine learning algorithms, descriptive statistical analysis was performed using IBM SPSS Statistics 27 software to examine the distribution of weight status categories across various sociodemographic, lifestyle, and dietary factors. Associations between categorical predictors and weight status categories were assessed using chi-square tests of independence, reported with *χ*^2^ statistics and *p*-values.

Following the descriptive analysis, various machine learning algorithms were used using Python 3.11, each serving different purposes depending on the properties of the dataset. Understanding the strengths and weaknesses of each model is essential for effective application in the health domains where accuracy is critical.

This study utilized the following models for prediction and factor analysis: support vector machines (SVM) (30), Random Forest (RF) (31), logistic regression (LR) (32, 33), gradient boosting (GB) (32), and decision tree (DT) (34). Voting and Stacking models, which are ensemble learning techniques used to improve predictive performance by combining multiple machine learning models, were also used. All seven of these models have proven track records in prediction, detection, management, and prevention applications across various areas of the health domain. These models were applied to the dataset at hand to predict each individual’s weight status as a measure of reliability. Then they ranked the feature importance of the predicted factors that most contributed to the classification result.

### 2.4 Optimization and validation

Each ML model has a unique set of model-specific parameters called hyperparameters. Fine-tuning their values is essential to optimizing model performance and enhancing predictive capabilities. Table 2 comprises a list of the hyperparameter values for the SVM, RF, LG, GB, and DT models.

**Table 2:** Hyperparameter optimization of machine learning models.

ML models	Hyperparameters	Value
SVM	Kernel type	‘rbf’
	Regularization parameter (C)	1.0
	Kernel-specific parameters (γ)	‘scale’
RF	Number of decision trees (n_estimators)	100
	Maximum depth of trees (max_depth)	10
	Number of features to consider for each split (max_features)	‘auto’
LG	Regularization strength (C)	1.0
	Penalty type	‘l2’
	Solver algorithms	‘lbfgs’
GB	Learning rate (*η*)	0.1
	Number of boosting stages (n_estimators)	100
	Maximum depth of individual trees (max_depth)	3
	Subsample fraction (subsample)	1.0
DT	Maximum depth of the tree (max_depth)	10
	Minimum samples required to split (min_samples_split)	2
	Minimum samples required at a leaf node (min_samples_leaf)	1

To validate the findings of the study, an exhaustive set of performance measures (metrics) was utilized along with k-fold cross-validation, a method that is widely recognized in healthcare research. Here, the 10-fold cross-validation approach (*k* = 10) was specifically used, where the dataset was divided into 10 equal parts. In each iteration, 90% of the data was used for training the model, and the remaining 10% served as the test set. This process was repeated 10 times, ensuring that every data record participated in the test set at least once. This method enhances the reliability of our models by ensuring thorough exposure to a variety of data scenarios, which enhances generalization prospects to unseen data.

Evaluating the classification performance of our models involved several metrics, each offering unique insights into their effectiveness. The primary tool for this assessment was the confusion matrix, which supported the visualization of performance by categorizing predictions into true positives, false positives, true negatives, and false negatives. Accuracy, one of the key metrics, was calculated as the ratio of correctly predicted observations to the total observations. While high accuracy indicates overall effectiveness, it might not always provide a complete picture, especially in imbalanced datasets. Therefore, other metrics were also considered, such as precision and sensitivity. Precision is crucial when minimizing false positives is a priority, while sensitivity (or recall) is vital for correctly identifying as many true positives as possible. In the context of the current study, ensuring that the model accurately identifies obese individuals (precision) while also capturing as many cases as possible (recall) was essential.

To balance the trade-off between sensitivity and recall, the F1-score was used to combine the two metrics. Given that different scenarios might require a stronger emphasis on either precision or recall, the F*β* score with β set at 0.5 was implemented, prioritizing precision slightly more than recall. Additionally, the Matthews correlation coefficient (MCC) was computed, offering a balanced measure of classification performance even in imbalanced datasets. MCC ranges from −1 to 1, where 1 indicates perfect prediction, 0 indicates random performance, and −1 indicates total disagreement between prediction and observation.

The MCC is defined as:


$$\mathrm{MCC}=\frac{\mathrm{TP}\times \mathrm{TN}-\mathrm{FP}\times \mathrm{FN}}{\sqrt{\left(\mathrm{TP}+\mathrm{FP})(\mathrm{TP}+\mathrm{FN})(\mathrm{TN}+\mathrm{FP})(\mathrm{TN}+\mathrm{FN}\right)}}$$

where TP, TN, FP, and FN represent true positives, true negatives, false positives, and false negatives, respectively.

Unlike accuracy, MCC considers all four confusion matrix categories and is thus considered a robust metric for evaluating binary and multiclass classification tasks, especially when the data are imbalanced (35).

Finally, the area under the receiver operating characteristic (ROC) curve, or AUC, was used to measure the model’s ability to distinguish between classes. AUC values range from 0.5 to 1, with higher values indicating greater discriminatory power. This metric is especially useful in understanding how well our model can differentiate between different weight statuses.

## 3 Results

### 3.1 Descriptive analysis

Table 3 presents an extensive analysis of the distribution of weight groups across various sociodemographic, lifestyle, and eating habit factors, revealing significant correlations among them. Each factor’s influence on weight status (underweight, healthy, overweight, or obese) is reflected in the percentages below and highlighted by the *Χ*^2^ (*p*-value), indicating statistical significance.

**Table 3:** Percentage distribution of weight status by sociodemographic, lifestyle, and eating habits.

Variable	Categories	Underweight *n* (%)	Normal *n* (%)	Overweight *n* (%)	Obese *n* (%)	*Χ*^2^ (*P*-value)
Family income	Low	406 (23.2)	357 (20.4)	455 (26)	531 (30.4)	74.4**
	Moderate	908 (22.2)	1,070 (26.2)	1,048 (25.6)	1,060 (25.9)	
	High	459 (36.5)	346 (27.5)	270 (21.5)	182 (14.5)	
Marital status	Single	1772 (26.6)	1726 (25.9)	1722 (25.8)	1,444 (21.7)	560.1**
	Married	1 (0.2)	47 (11)	51 (11.9)	329 (76.9)	
Living status	With family	1743 (25.6)	1,681 (24.7)	1,693 (24.9)	1,682 (24.7)	49.4**
	With a Roommate	30 (10.2)	92 (31.4)	80 (27.3)	91 (31.1)	
Academic level	Student	1,518 (27.1)	1,447 (25.8)	1,357 (24.2)	1,288 (23)	72.3**
	Bachelor’s degree	255 (17.2)	326 (22)	416 (28.1)	485 (32.7)	
Smoking	No	1,677 (25.2)	1,647 (24.7)	1,627 (24.4)	1708 (25.6)	0.7
	Yes	96 (22.2)	126 (29.1)	146 (33.7)	65 (15)	
Physical activity	Active	1,190 (24.1)	1,087 (22)	1,324 (26.8)	1,335 (27)	75.2**
	Inactive	583 (27)	686 (31.8)	449 (20.8)	438 (20.3)	
Sleeping hours	6–8 h	1,196 (24.4)	1,342 (27.4)	1,250 (25.5)	1,109 (22.6)	17.6**
	< 5 h	577 (26.3)	431 (19.6)	523 (23.8)	664 (30.3)	
Food allergy	No	1,314 (23.8)	1,382 (25)	1,186 (21.5)	1,642 (29.7)	80.7**
	Yes	459 (29.3)	391 (24.9)	587 (37.4)	131 (8.4)	
Regular lunch	No	1,216 (24.4)	1,200 (24.1)	1,202 (24.2)	1,356 (27.3)	5.8*
	Yes	557 (26.3)	573 (27.1)	571 (27)	417 (19.7)	
Regular dinner	No	519 (18.6)	772 (27.6)	737 (26.4)	765 (27.4)	42.2**
	Yes	1,254 (29.2)	1,001 (23.3)	1,036 (24.1)	1,008 (23.4)	
Daily breakfast	Yes	1,014 (24.2)	1,094 (26.1)	1,030 (24.6)	1,046 (25)	3
	No	759 (26.1)	679 (23.3)	743 (25.6)	727 (25)	
Breakfast on the weekend	Yes	1,246 (24.9)	1,297 (25.9)	1,352 (27)	1,119 (22.3)	94.5**
	No	527 (25.4)	476 (22.9)	421 (20.3)	654 (31.5)	
Water quantity	Normal	1,515 (29.9)	1,334 (26.3)	1,073 (21.2)	1,145 (22.6)	257.2**
	A lot	258 (12.7)	439 (21.7)	700 (34.6)	628 (31)	
Eating speed	Normal	1,186 (27.9)	1,047 (24.6)	1,065 (25.1)	952 (22.4)	17.3**
	Quick eater	587 (20.7)	726 (25.5)	708 (24.9)	821 (28.9)	
Water drink	Normal	940 (20.9)	1,179 (26.2)	1,285 (28.6)	1,093 (24.3)	74**
	Low	833 (32.1)	594 (22.9)	488 (18.8)	680 (26.2)	
Added salt	Always	1,152 (31.5)	789 (21.6)	829 (22.7)	888 (24.3)	292.1**
	Sometimes	453 (21.1)	616 (28.7)	601 (28)	479 (22.3)	
	Rarely	168 (13.1)	368 (28.6)	343 (26.7)	406 (31.6)	
Added sugar	Always	520 (24.1)	509 (23.6)	570 (26.5)	555 (25.8)	9.5*
	Sometimes	826 (25.9)	796 (24.9)	764 (23.9)	808 (25.3)	
	Rarely	427 (24.5)	468 (26.8)	439 (25.2)	410 (23.5)	
Added oil	Always	514 (22.2)	449 (19.4)	702 (30.3)	655 (28.2)	348.4**
	Sometimes	638 (21.1)	831 (27.5)	750 (24.8)	805 (26.6)	
	Rarely	621 (35.5)	493 (28.2)	321 (18.4)	313 (17.9)	

*Statistically significant: *p ≤ 0.05 and **p ≤ 0.001.

Individuals from low-income families show a higher prevalence of obesity (30.4%) compared to being underweight (23.2%). Conversely, those with high family incomes are more likely to be underweight (36.5%) and less likely to be obese (14.5%). This trend, noticeable by a significant (*Χ*^2^ = 74.4, *p* = 0.001), suggests a strong correlation between income levels and weight status. It highlights how economic factors can influence dietary choices and lifestyle habits, ultimately affecting weight. The impact of marital status is also noteworthy. Among single individuals, the distribution across weight statuses is relatively even, with a slight disposition toward being underweight (26.6%). In contrast, married individuals exhibited a significantly higher rate of obesity (76.9%). This difference, confirmed by a high (*Χ*^2^ = 560.1, *p* = 0.001), suggests the potential influence of marital life on lifestyle choices that affect weight, such as diet and physical activity.

Regarding living status, those residing with family have an even distribution across weight statuses. In comparison, individuals living with roommates show higher percentages of obesity. This observation is statistically significant (*Χ*^2^ = 49.4, *p* = 0.001) and may reflect differences in dietary habits and social influences on eating behaviors in different living arrangements.

Students are more likely to be underweight or have a normal weight, while individuals with a bachelor’s degree tend to be overweight or obese. This pattern (*Χ*^2^ = 72.3, p = 0.001) could be attributed to post-education lifestyle changes, including work-related sedentary behavior and altered eating habits.

Interestingly, smoking habits do not show a significant correlation with weight status (*Χ*^2^ = 0.7, *p* = 0.415), suggesting that factors other than smoking are more influential in determining weight. On the other hand, physical activity levels have a noticeable effect. Active individuals are more likely to be overweight or obese, while inactive individuals tend toward a normal weight (*Χ*^2^ = 75.2, *p* = 0.001). This suggests that while physical activity is crucial for health, it is not the sole determinant of weight status and must be balanced with other factors such as diet. Sleep patterns contribute significantly to weight status. Individuals getting 6–8 h of sleep show a balanced weight distribution, whereas those sleeping less than 5 h are more prone to obesity (*Χ*^2^ = 17.6, *p* = 0.001). This finding emphasizes the importance of adequate sleep in maintaining a healthy weight and suggests that sleep deprivation may lead to lifestyle choices that promote obesity.

Food allergies are significantly associated with weight status, where the absence of allergies correlates with a higher percentage of obesity (*Χ*^2^ = 80.7, *p* = 0.001). This might be due to dietary restrictions in individuals with food allergies, resulting in different food choices that affect weight. Meal regularity also influences weight status; irregular patterns in lunch and dinner are associated with higher obesity rates (*Χ*^2^ = 5.8, *p* = 0.016 for lunch; *Χ*^2^ = 42.2, *p* = 0.001 for dinner). This highlights the role of consistent eating patterns in weight management. Water consumption habits and eating speed further contribute to the observed trends in weight status. Quick eating is also correlated with a higher likelihood of obesity (*Χ*^2^ = 17.3, *p* = 0.001), suggesting the importance of eating pace as a factor of weight control.

### 3.2 Machine learning analysis

The results in Table 4 demonstrate the effectiveness of various algorithms across the following three weight categories: underweight, overweight, and obese. The primary metrics for evaluation are accuracy, F1 score, and Matthews correlation coefficient (MCC), which provides a balanced view of each model’s performance.

**Table 4:** Machine learning performance analysis for the holistic personalized weight status prediction.

Model	AUC	Accuracy	F1	Precision	Recall	MCC
Underweight						
RF	0.950	**94.6**	0.92	0.93	0.91	0.89
SVM	0.931	92.8	0.91	0.9	0.92	0.88
GB	0.892	89.5	0.88	0.87	0.89	0.85
LR	0.761	76.5	0.76	0.75	0.78	0.72
DT	0.803	80.7	0.8	0.81	0.79	0.76
Voting	0.935	93.1	0.93	0.92	0.94	0.9
Stacking	0.928	92.4	0.91	0.9	0.92	0.88
Overweight						
RF	0.945	**90.3**	0.9	0.89	0.91	0.87
SVM	0.922	88.9	0.89	0.88	0.9	0.85
GB	0.877	85.2	0.85	0.84	0.86	0.82
LR	0.743	74.1	0.74	0.73	0.75	0.7
DT	0.797	78.9	0.79	0.78	0.8	0.76
Voting	0.925	90.1	0.92	0.91	0.93	0.88
Stacking	0.913	89.4	0.9	0.89	0.91	0.86
Obesity						
RF	0.971	**96.8**	0.96	0.97	0.95	0.93
SVM	0.957	95.3	0.95	0.94	0.96	0.92
GB	0.928	92.7	0.92	0.91	0.93	0.89
LR	0.799	79.2	0.8	0.79	0.81	0.77
DT	0.831	83.4	0.83	0.82	0.84	0.8
Voting	0.962	95.7	0.95	0.96	0.94	0.91
Stacking	0.959	95.4	0.94	0.93	0.95	0.9

Within the underweight category, the RF model demonstrates superior performance with the highest accuracy (94.6%), F1 score (0.92), and MCC (0.89). The SVM model also shows positive results, achieving an accuracy of 92.8%, an F1 score of 0.91, and an MCC of 0.88, though it does not live up to the RF model. The GB, LR, and DT models exhibit varying levels of effectiveness, with LR performing the least effectively in comparison to RF and SVM. The Voting and Stacking models perform closely to RF, with Voting achieving an accuracy of 93.1% and an MCC of 0.90, while Stacking achieves an accuracy of 92.4% and an MCC of 0.88.

In the overweight category, the RF model maintains strong performance, leading in accuracy (90.3%) and MCC (0.87). However, the performance in the overweight category is lower than in the underweight and obese categories, where the RF model achieves higher accuracy. The performance gap between the RF and SVM models, which achieve an accuracy of 88.9% and an MCC of 0.85, is smaller compared to the underweight category. GB (accuracy of 85.2%, MCC of 0.82) also performs well, though LR and DT remain less effective in this group. The Voting model performs well in the overweight category, achieving an accuracy of 89.4%, an F1 score of 0.90, and an MCC of 0.86.

For the obese category, RF again performs best, achieving an accuracy of 96.8%, an F1 score of 0.96, and an MCC of 0.93. SVM shows strong performance with an accuracy of 95.3% and an MCC of 0.92, while GB also performs well with an accuracy of 92.7% and an MCC of 0.89. While LR and DT perform better in this category compared to the overweight group, they still fall short of the top models. LR achieves an accuracy of 79.2% with an MCC of 0.77, while DT scores an accuracy of 83.4% and an MCC of 0.80.

The RF model consistently exhibits the highest performance across all three groups for accuracy, F1 score, and MCC, demonstrating its robustness and suitability for weight status prediction across different categories. However, RF’s superiority varies among weight groups. While it is significantly ahead in the underweight group, in the overweight and obese categories, other models such as Stacking, Voting, and GB perform better. This highlights the importance of model selection based on specific application needs in predictive analytics.

### 3.3 AUC comparison

**Figure 1:**
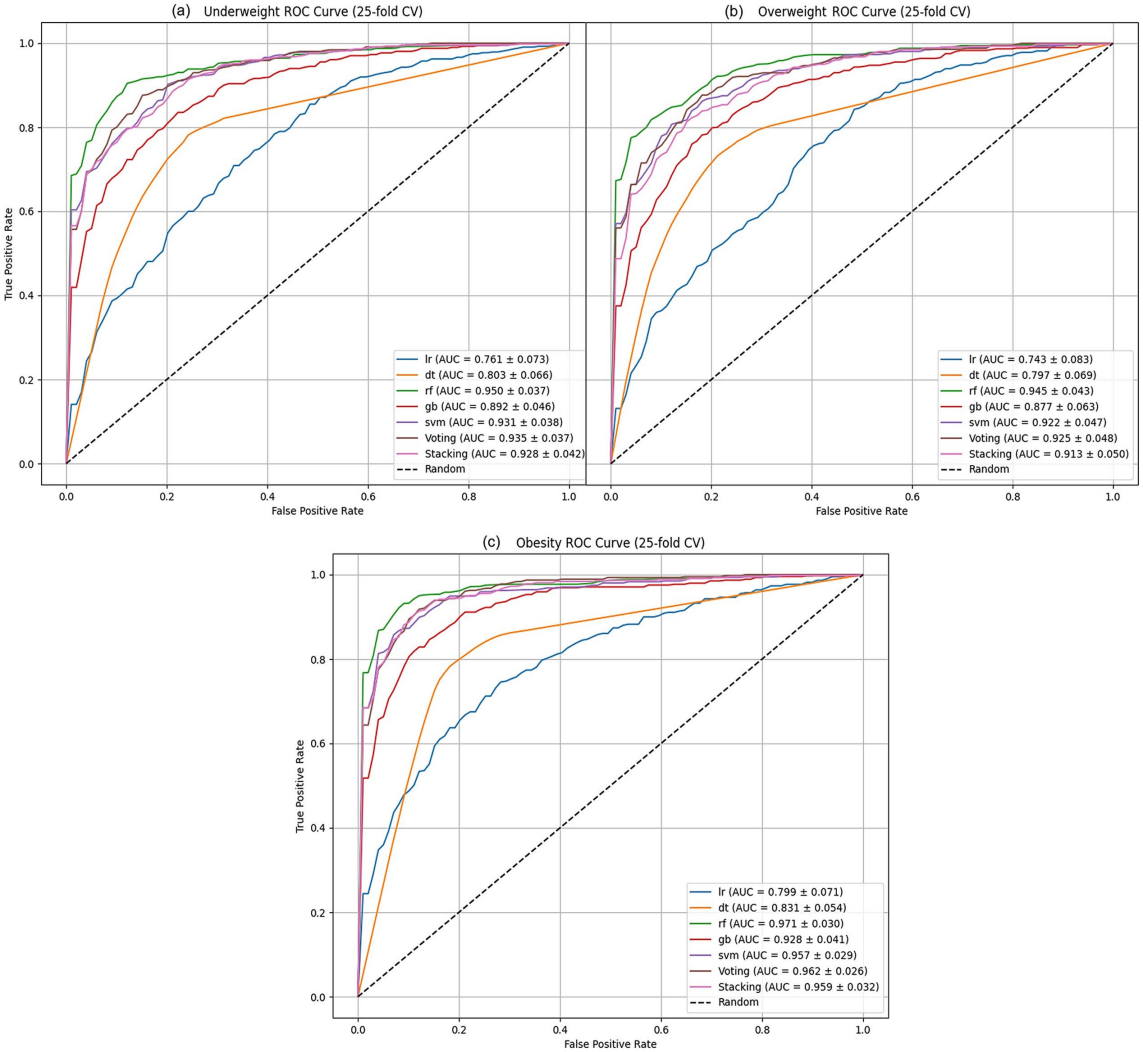
ML algorithms AUC performance analysis of: **(a)** underweight, **(b)** overweight models, and **(c)** obesity.

The AUC results, shown in Figures 1a–c, illustrate how well the models perform across different weight categories. In the underweight group (Figure 1a), the RF model leads with an AUC of 0.95, demonstrating strong classification ability. The SVM follows closely with an AUC of 0.931, also showing good performance, though slightly behind RF. Other models, such as GB and the ensemble methods (Voting and Stacking), perform reasonably well but fall behind RF and SVM. Logistic regression (LR) and decision tree (DT) models show the weakest performance in this category.

In the overweight group (Figure 1b), the RF model continues to perform best, with an AUC of 0.945. However, the difference between RF and SVM (AUC = 0.922) is smaller than in the underweight group. The GB model, while still behind RF and SVM, performs moderately well with an AUC of 0.877. Voting (AUC = 0.925) also performs better than GB, while DT and LR remain less effective in classifying overweight cases.

For the obese group (Figure 1c), RF once again leads with an AUC of 0.971, the highest among all categories. The SVM and GB models show solid performance, with AUCs of 0.957 and 0.928, respectively. Ensemble models such as Voting (AUC = 0.962) and Stacking (AUC = 0.959) show competitive results, closely following SVM. DT and LR improve their performance in this group, but still lag behind the top models.

The RF model consistently shows the highest AUC across all categories, indicating its strong classification performance. Other models, such as SVM and ensemble methods, show more competitive results in the overweight and obese categories, suggesting that the best model can vary depending on the weight group being analyzed. These findings highlight the importance of selecting the appropriate model based on the specific context of the data.

### 3.4 Features importance

**Figure 2:**
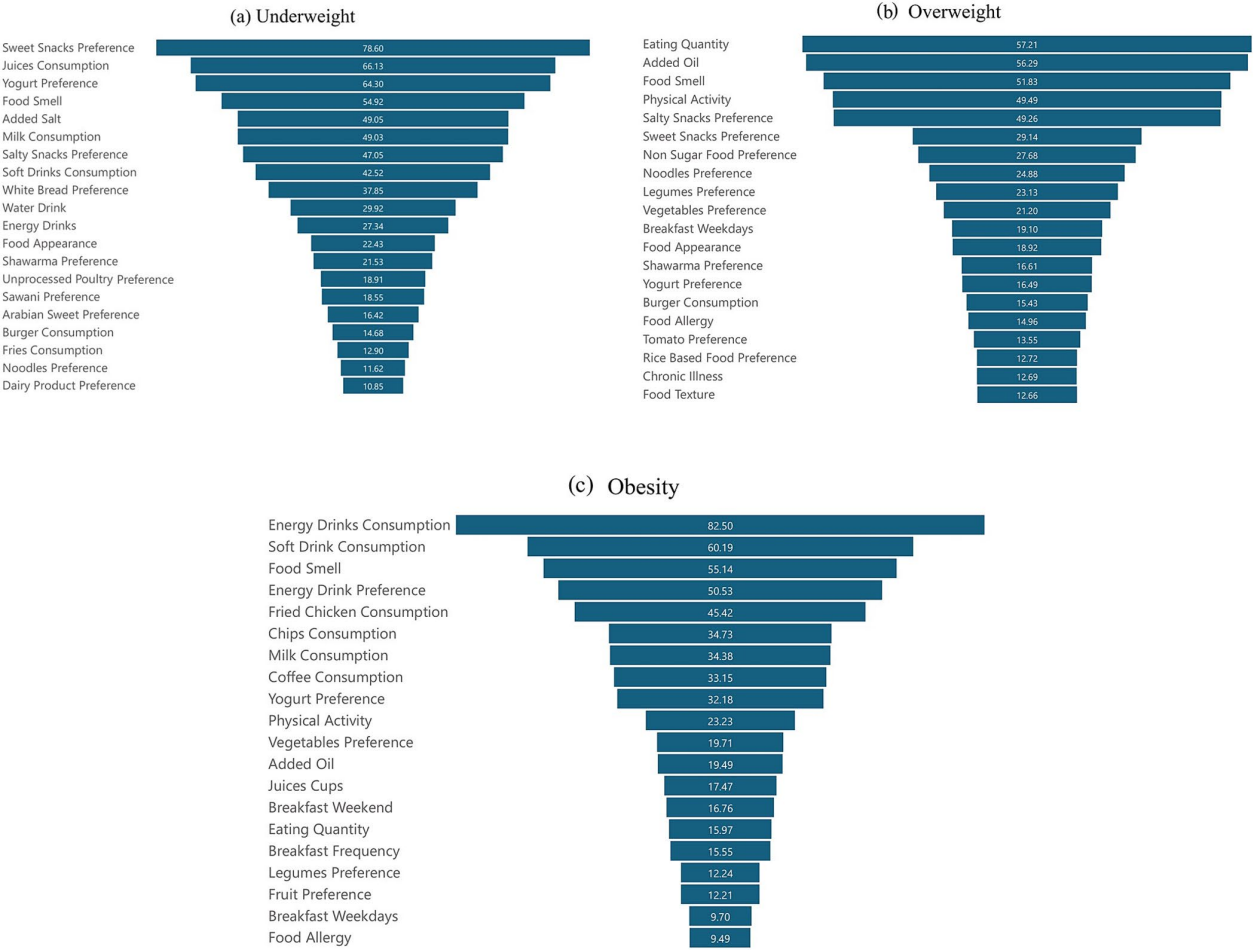
ML algorithms features importance analysis of: **(a)** underweight, **(b)** overweight, and **(c)** obesity, and the average importance of the top 20 variables ranked according to their level of causality in weight status.

The results in Figure 2 illustrate RF feature importance ranking for the underweight, overweight, and obesity categories. Dietary preferences such as sweet snacks, juice consumption, and yogurt preference are top drivers of underweight status, alongside food smell and a tendency toward added salt. Milk consumption and preferences for salty snacks, soft drinks, and white bread also play a significant role in this category. Food appearance and preferences, unprocessed poultry preference, and Sawani preference are important. Additionally, water consumption, energy drinks, Arabian sweet preference, burger consumption, fries consumption, noodles preference, and dairy product preference are also significant factors.

In the overweight group, the quantity of food is the most crucial feature, highlighting the impact of portion sizes on weight. This is closely followed by added oil, which highlights the significance of cooking habits. Other important factors include food smell and physical activity, reflecting lifestyle influences. Preferences for salty snacks, sweet snacks, non-sugary food, noodles, legumes, vegetables, and breakfast on weekdays indicate specific eating patterns that are significant for predicting overweight status. Food appearance, shawarma preference, yogurt preference, burger consumption, food allergies, tomato preference, rice-based food preference, chronic illness, and food texture contribute to understanding dietary choices and their effects on weight.

On the other hand, personal life circumstances, such as the consumption of energy drinks and soft drinks, are also significant contributors to obesity. These are followed by food smell and energy drink preferences, reflecting dietary habits. The consumption of fried chicken, chips, milk, and coffee also emerges as significant, with yogurt preference, physical activity, vegetable preference, and added oil indicating specific dietary influences. Other important factors include juice consumption, breakfast habits and frequency, eating quantity, legume preference, fruit preference, and food allergies.

## 4 Discussion

This research aimed to develop an understanding of how different lifestyles and dietary behaviors impact weight status, utilizing machine learning to explore the complex non-linear relationships between these factors and weight. Traditional statistical analysis methods are effective but often rely on assumptions of linearity and predefined relationships. In contrast, machine learning can detect intricate, non-linear patterns and interactions, offering a more insightful understanding of the combined effects of multiple predictors on weight status and stability.

The findings in Table 3 highlight the intricate interplay of sociodemographic factors, lifestyle choices, and eating habits with weight status. For example, the study reveals that low-income families tend to have higher obesity rates, while high-income families are more likely to be underweight. These results are consistent with research by Drewnowski and Specter (36), which identifies socio-economic status as a key determinant of dietary choices and health outcomes. Sociodemographic factors such as family structure and living arrangements significantly influence weight status, aligning with studies by Moore et al. that emphasize the role of social dynamics in health behaviors (1). These insights suggest that personalized interventions considering such factors could improve weight management strategies and overall health outcomes.

A key contribution of this study is addressing the multifactorial nature of weight status. While conventional analyses can highlight individual predictors, they fail to capture the interactions and cumulative effects of multiple variables. Our machine learning models, incorporating algorithms such as gradient boosting (GB), Random Forest (RF), logistic regression (LR), and ensemble methods (Voting, Stacking), overcome this limitation. It allows for a deeper, more accurate prediction of weight status by analyzing how different factors interact to influence weight.

The superior performance of the RF model across all weight categories, as demonstrated by accuracy, F1 score, and Matthew’s correlation coefficient (MCC), highlights its robustness and reliability. RF’s ability to consistently outperform other models makes it a strong candidate for weight status prediction in diverse populations. The relatively excellent performance of models such as GB and support vector machines (SVM) suggests that different algorithms may be better suited for specific weight groups. For example, while RF generally provides the most accurate results, GB and SVM show competitive performance in the overweight and obese groups, suggesting that model selection should consider the specific context of the analysis.

This study aligns with the broader trend toward personalized health solutions, emphasizing the need for individualized healthcare. The ability to choose the most appropriate model for a given population or condition ensures that interventions are more effective and tailored to specific needs. This personalized approach not only improves treatment outcomes but also enhances the overall efficiency of healthcare systems.

Comparing our findings with previous studies, it can be seen that RF consistently outperforms other algorithms in weight status prediction, aligning with the results of research by Elias et al. (37) and Rahman et al. (38), where RF demonstrated strong predictive power in obesity-related studies. While other models, such as SVM and GB, offer strong predictive capabilities, RF’s superior interpretability and consistency in performance make it particularly valuable for developing healthcare interventions. By capturing the interactions among various lifestyle, dietary, and sociodemographic factors, machine learning models provide a deeper understanding of the factors contributing to health outcomes, an essential step in advancing personalized medicine.

The Random Forest (RF) model demonstrated consistently high classification performance, as evidenced by area under the curve (AUC) metrics that were particularly strong in the underweight category, nearing values close to 1.0. This finding reaffirms the suitability of ensemble learning models such as RF for imbalanced classification tasks, where class-specific optimization is critical. Similar observations have been reported by Pérez-Cruzado et al., who showed that ensemble approaches outperformed traditional models in identifying obesity risk factors, especially when AUC was used as the primary performance metric (39). This emphasizes the need to tailor model selection based on the weight category being analyzed, as no single model uniformly excels across all categories.

Feature importance analysis within the RF model offered valuable insights into the varying determinants of weight status among students. These determinants were not only quantitatively distinct but also contextually shaped by socio-demographic, psychological, and dietary variables.

For underweight students, the model identified high consumption of specific dietary items, such as white bread, diet foods, and added salt, as a significant factor. More importantly, non-nutritional factors such as depressive symptoms, larger family size, and milk consumption habits at home also emerged as strong predictors. These findings are consistent with the study of Güvenç and Bulut, who highlighted how emotional wellbeing and family dynamics significantly impact adolescent nutritional status (40). Similarly, Blaine established that depression can lead to weight loss due to appetite suppression, demonstrating that mental health plays a crucial role at both ends of the weight spectrum (41).

In the overweight group, different variables took precedence. Cooking habits, particularly the use of added oils, as well as meal volume, income level, and hydration (water consumption), were influential. These results align with Alakaam et al., who found that cultural food preparation methods, such as frying and excessive oil use, contributed to increased body weight in diverse populations (42). Moreover, the association between low water intake and increased caloric density has been documented as a contributing factor in weight gain, supporting our model’s outputs (43).

Among students classified as obese, the strongest predictors were frequent consumption of energy drinks and sweetened beverages, along with sensitivity to the sensory appeal of food, such as smell, appearance, and taste. This aligns with studies by Puhl et al., who showed that obese individuals exhibit heightened neural responses to food-related cues, especially those with strong sensory attributes (44). Similarly, Azagba et al. found a positive correlation between high school students’ BMI and their frequency of energy drink consumption, emphasizing the obesogenic potential of these beverages (45).

Furthermore, our findings on income and food choices showed that the “food insecurity-obesity paradox” is consistent with the findings by Dinour et al. (46), where limited resources lead to the selection of inexpensive, calorie-dense foods, increasing obesity risk. This reinforces the idea that economic context plays a pivotal role in shaping dietary behavior and weight outcomes.

The RF model’s superior performance in this study, reflected in its higher accuracy and feature importance rankings, solidifies its role as a powerful tool for predicting weight status, as shown in Figure 2b. Its ability to handle a diverse range of predictors, along with its clear feature importance rankings, makes it especially suitable for this analysis.

While other models, such as support vector machines (SVM), gradient boosting, and ensemble methods, performed well, the RF model consistently outperformed them in terms of accuracy and interpretability. For instance, although SVM and gradient boosting showed competitive performance in terms of accuracy, they lacked the intuitive feature importance insights offered by RF. This interpretability is crucial for understanding the impact of dietary habits and consumption patterns on weight status. The RF model, due to its balance of accuracy and interpretability, was the most appropriate choice for this study. Regardless, each model has its place depending on the context, and other models may provide valuable predictive capabilities in different applications.

## 5 Study limitations

This study explored how diet, lifestyle, and background factors relate to weight status among female university students in Palestine and the UAE. Using machine learning, we identified unique patterns for underweight, overweight, and obesity that traditional methods might miss. While tools such as SMOTE improved model balance, they may have introduced data that does not fully reflect real life. Self-reported answers also carry the risk of recall bias. Since the study is cross-sectional, we cannot say what causes what. Future research should include things such as hormones, menstrual health, and genetics to better understand what drives weight differences and help create more personalized support for young women.

## 6 Conclusion

This study showcased the prowess of machine learning models as statistical tools in identifying the interaction between the complex, interconnected variables of weight status. Factors, including depression severity and family income, were prominent components of the overweight variable set. Marital status and food allergies were key for the obese group, and bread and diet food preferences were the most notable for the underweight. Eating quantity had an impact that varied by weight group. Moreover, factors including food smell and milk consumption were important all across, while others, including energy drink consumption and added oil, varied more. Psychological elements such as depression and family-related details, such as the number of people in the household, were not only part of our data but also significantly associated with being underweight.

Based on our findings, it is clear that universities can play a stronger role in supporting students’ health. Providing practical education on eating habits, physical activity, and sleep can make a significant difference. Creating a campus environment where healthy food is both available and affordable is also key. Personalized support through counseling, along with tools to help spot students who may be at risk, can ensure timely help and promote healthier lifestyles overall.

The findings of this study indicate the need for future research that focuses on developing healthier weight management strategies, with a particular emphasis on obesity management, reversibility, and prevention.

## Data Availability

The raw data supporting the conclusions of this article will be made available by the authors, without undue reservation.
